# Synaptic-immune interactions in neuropathic pain: complement-mediated remodeling of spinal dorsal horn microcircuits

**DOI:** 10.3389/fcell.2026.1798053

**Published:** 2026-04-09

**Authors:** Wen-ming Zhou, Kai Zhang, Wei-wei Ma, Yong-qiang Shi, Yan-Bo Dong, Zi-yu Yuan, Hai-hong Zhang

**Affiliations:** 1 Department of Orthopedics, The Second Hospital and Clinical Medical School, Lanzhou University, Lanzhou, Gansu, China; 2 Orthopaedics Key Laboratory of Gansu Province, The Second Hospital of Lanzhou University, Lanzhou, Gansu, China; 3 Department of Orthopedics, Xi’an Honghui Hospital, Xi’an, China

**Keywords:** central sensitization, complement system, inhibitory synapses, neuropathic pain, synaptic remodeling

## Abstract

Neuropathic pain (NP) has long been considered to arise primarily from abnormal neuronal excitability and ion channel dysfunction. However, accumulating evidence indicates that structural and functional remodeling of microcircuits in the spinal dorsal horn (SDH) also plays a critical role in the initiation and maintenance of pain. This article reviews and integrates recent findings demonstrating that aberrant activation of the complement system in the mature central nervous system preferentially targets inhibitory synapses and their microenvironment through microglia-mediated synaptic pruning. Disruption of perineuronal nets, remodeling of synaptic surface glycocodes, and activity-dependent complement deposition collectively increase the vulnerability of inhibitory circuits to immune-mediated elimination. These processes interact with alterations in ionic homeostasis, including downregulation of KCC2 and polarity shifts in GABAergic transmission, thereby promoting disinhibition, central sensitization, and impairment of sensory gating. Importantly, complement-driven synaptic remodeling is not an isolated inflammatory event but rather the outcome of coordinated regulation within a neuron–glia–immune network. This perspective helps explain the limited efficacy of neuron-centered analgesic strategies and provides a theoretical basis for the development of disease-modifying therapies aimed at synaptic preservation and stabilization of the synaptic microenvironment.

## 1 Introduction

Neuropathic pain (neuropathic pain, NP) is defined by the International Association for the Study of Pain (IASP) as pain caused by a lesion or disease of the somatosensory system, and is characterized by persistent or paroxysmal pain, sensory abnormalities, and hyperalgesia (Treede et al., 2008; Sommer, 2025). Epidemiological studies indicate that NP affects approximately 7%–10% of the global population and is frequently associated with substantial functional impairment and multiple comorbidities, thereby constituting a significant public health burden (Sommer, 2025). Although opioids and gabapentinoids are widely used in clinical practice, their therapeutic efficacy is limited in most patients, and long-term use is often accompanied by the development of tolerance and adverse effects, which markedly restricts their sustained clinical benefit (Soliman et al., 2025). This limited treatment efficacy partly reflects a long-standing “neuron-centric view” of NP pathogenesis, which primarily emphasizes aberrant ion channel expression or dysregulated neurotransmitter release, while comparatively neglecting the influence of the neuronal microenvironment and its structural and functional states (Sharma et al., 2025).

With the rapid adoption of high-resolution imaging techniques and single-cell transcriptomics in neuroscience research, the understanding of NP pathophysiological mechanisms has in recent years expanded from the traditional “neuron-centric view” to levels involving the neural microenvironment and glia-driven processes. Microglia are no longer regarded merely as supportive cells; rather, following peripheral injury, they act as active participants that respond to changes in neuronal activity and remodel neuronal signaling pathways through microenvironmental sensing (Malcangio et al., 2025). Peripheral nerve injury induces dynamic alterations in the state of microglia in the spinal dorsal horn (SDH), including changes in cell number, transcriptional states, and signaling molecule expression, all of which are closely associated with NP-related behavioral manifestations (Inoue and Tsuda, 2018). Microglia–neuron interactions have been shown to regulate neuronal excitability through P2X4 receptor–dependent mechanisms, for example, via the release of brain-derived neurotrophic factor (BDNF), thereby contributing to the maintenance of pain sensitization (Inoue, 2024). Meanwhile, high-throughput single-cell sequencing technologies are revealing glial heterogeneity in the spinal cord and dorsal root ganglia, providing molecular details and lineage-level perspectives for understanding glial state transitions under NP-associated microenvironmental conditions (Masuda et al., 2020). When combined with existing functional neural network imaging approaches, these technologies provide a new evidentiary basis for elucidating how glial cells participate in SDH synaptic plasticity and their roles in NP pathology.

Within this complex network of cellular interactions, aberrant activation of the innate immune system is considered a key mechanism linking peripheral nerve injury to remodeling of central neural circuits. Among these processes, the complement cascade, a highly conserved component of the innate immune system, is no longer viewed as functioning solely in immune defense within the central nervous system. Classical studies have demonstrated that during critical periods of nervous system development, complement components C1q and C3 localize to synaptic structures and participate in the refinement of neural circuits by mediating microglia-dependent synaptic elimination. However, whether the same molecular framework operates in the adult central nervous system remains an open question. In the mature CNS, complement activation is likely to occur under different regulatory conditions and may involve additional modulatory mechanisms that distinguish pathological synaptic elimination from developmental pruning (Stevens et al., 2007). Studies by Yousefpour et al. (Yousefpour et al., 2025) indicate that under neuropathic pain conditions, this complement-dependent synaptic pruning mechanism, which normally operates during development, becomes aberrantly reactivated in the mature central nervous system. Peripheral nerve injury can enhance complement signaling within the spinal dorsal horn, thereby driving microglia-mediated removal of inhibitory synapses, leading to structural and functional remodeling of central neural circuits and promoting the maintenance of pain sensitization. These findings suggest that complement-mediated synaptic regulation is not confined to developmental stages but can be misappropriated under pathological conditions and transformed into a mechanism that facilitates disease progression.

Under pathological conditions, complement-mediated pathological reprogramming may shift the function of the complement system from physiological synaptic refinement toward the preferential elimination of specific inhibitory synapses within the spinal dorsal horn (SDH). Evidence indicates that complement components deposit at synapses in the SDH and are accompanied by microglia-mediated synaptic removal, with the loss of inhibitory synapses occurring earlier than that of excitatory synapses, suggesting selective vulnerability of inhibitory connections (Yousefpour et al., 2025). The reduction of inhibitory synapses alters the local excitatory–inhibitory (E/I) balance within the SDH and represents a core mechanism underlying central sensitization and tactile allodynia in NP (Malcangio et al., 2025; Ji et al., 2013). Computational modeling and electrophysiological studies further indicate that such E/I imbalance and disinhibition help explain the emergence and persistence of allodynia (Ginsberg et al., 2025; Gong et al., 2019).

Complement-mediated synaptic elimination is a process characterized by precise spatiotemporal regulation rather than a simple, isolated phagocytic event. During development, components of the classical complement pathway (C1q/C3) are localized to synapses and participate in selective pruning of neural circuits, a mechanism that has been experimentally validated (Stevens et al., 2007); in the mature central nervous system, aberrant complement activation can also reinitiate synaptic remodeling. Perineuronal nets (PNNs) are dense extracellular matrix structures that enwrap inhibitory interneurons and are essential for synaptic homeostasis and the function of inhibitory networks; alterations in PNN density and architecture are associated with abnormal synaptic plasticity (Totaro et al., 2025). Sialic acid moieties within the synaptic surface glycocalyx can effectively inhibit the binding of complement component C1q, thereby reducing complement-mediated synaptic loss (Linnartz et al., 2012). Although no single study has yet provided a unified causal framework encompassing all of these mechanisms, the available high-level evidence collectively supports a pathological link between complement activation and multilayered regulation of the synaptic microenvironment. Accordingly, this article focuses on immune–synapse interactions and systematically examines the pathological relationship between complement activation and neural circuit remodeling.

## 2 Structural basis and immune surveillance functions of the synaptic microenvironment in the spinal dorsal horn

**FIGURE 1:**
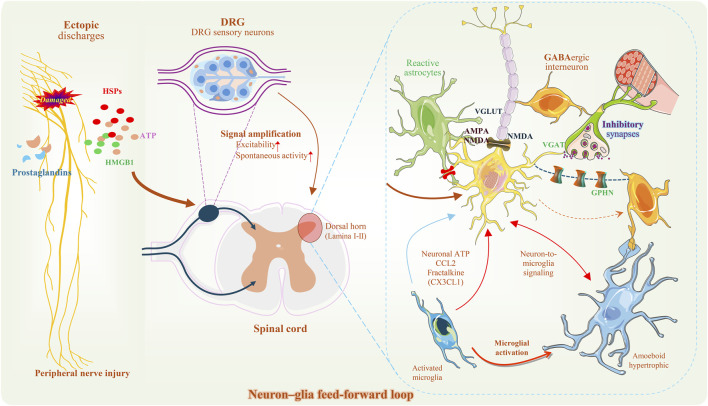
Schematic illustration of sensory pathway input and neuron–glia microcircuits in the spinal dorsal horn following peripheral nerve injury. Peripheral nerve injury and aberrant firing; release of injury-associated molecules; increased excitability of DRG sensory neurons; projection of sensory axons to the spinal dorsal horn (Lamina I–II). Local microcircuit architecture in the spinal dorsal horn: excitatory projection neurons, GABAergic inhibitory interneurons, microglia, and reactive astrocytes. Composition of excitatory and inhibitory synapses and associated markers (VGLUT, VGAT, GPHN); glutamate receptor types (AMPA, NMDA). Neuron–glia signaling molecules (ATP, CCL2, CX3CL1); microglial morphological states (ramified, hypertrophic). (Some graphical elements were adapted and modified from Servier Medical Art (https://smart.servier.com), licensed under a Creative Commons Attribution 3.0 Unported License).

The spinal dorsal horn (spinal dorsal horn, SDH), as the first central relay for somatosensory information processing, relies critically on the precise regulation of synaptic transmission to ensure functional fidelity (Todd, 2010). However, during the pathological progression of chronic pain, synaptic structures and local circuits undergo large-scale and directionally biased remodeling, a phenomenon that extends beyond the explanatory scope of the traditional view of synapses as binary information units composed solely of presynaptic and postsynaptic membranes (Kuner and Flor, 2016). Building on the concept of the “tripartite synapse,” contemporary synaptic biology has further proposed a multicomponent model of synaptic architecture, in which the functional synaptic entity comprises not only the presynaptic terminal and postsynaptic specializations, but also the surrounding glial processes and the perisynaptic extracellular matrix (ECM) (Araque et al., 1999; Dityatev et al., 2010). Within this highly organized subcellular microenvironment, immune-related elements—particularly microglia and microglia-mediated complement signaling—are not exogenous inflammatory factors, but rather endogenous components that persist over time and participate in the regulation of synaptic structure and function (Figure 1).

### 2.1 Reactivation of developmental synaptic pruning mechanisms under pathological conditions

Synaptic elimination is not a pathological process specific to NP, but rather a fundamental biological event during nervous system development (Faust et al., 2021). During early postnatal critical periods, the central nervous system selectively removes excessively generated and functionally weak synapses through activity-dependent mechanisms, thereby achieving refinement of neural circuit connectivity and functional optimization; this process is commonly referred to as synaptic pruning (Faust et al., 2021; Hensch, 2005). A large body of developmental studies indicates that this process is characterized by synaptic competition and experience-dependent regulation and represents a basic prerequisite for the formation of mature neural networks, rather than an abnormal phenomenon under pathological conditions (Hata et al., 2009).

Notably, under NP conditions, the spinal dorsal horn (SDH) exhibits features resembling a “developmental reversal” analogous to those observed during developmental stages. Transcriptomic studies indicate that nerve injury can induce the reactivation of a subset of embryonic or juvenile molecular programs in the adult sensory system, including pathways related to axonal growth, regulation of phagocytosis, and immune-associated signaling (Costigan et al., 2009). In parallel, complement cascade signaling, which is normally maintained at low expression levels after the completion of development—particularly the C1q–C3 pathway involved in the regulation of synaptic phagocytosis—is reactivated and markedly upregulated under NP conditions (Stevens et al., 2007; Yousefpour et al., 2025).

Although these processes share molecular similarities with developmental synaptic pruning, their biological significance differs fundamentally in functional orientation. Developmental synaptic pruning is a constructive process aimed at eliminating redundant connections and improving the signal-to-noise ratio of neural circuits (Faust et al., 2021); by contrast, synaptic elimination under NP conditions manifests as a pathological event, with inhibitory synapses that subserve sensory gating functions often constituting the primary targets, thereby directly disrupting circuit stability and inducing disinhibition and central sensitization (Coull et al., 2005). This shift from physiological refinement to pathological structural loss suggests a breakdown of regulatory mechanisms that maintain immune tolerance and synaptic homeostasis in the mature nervous system, accompanied by a transition of microglia from homeostatic maintenance to aberrant activation states, which is considered a key feature of neuropathological alterations (Whalley, 2017).

### 2.2 Functional transition of microglia from synaptic surveillance to structural stripping

Within the multidimensional architecture of the synaptic microenvironment, microglia serve as the principal surveillance cells of central immune homeostasis and, together with the classical tripartite synapse model and the extracellular matrix, participate in maintaining the dynamic stability of neural circuits. Homeostatic microglia are characterized by highly dynamic ramified processes that continuously extend and retract, forming transient contacts with presynaptic and postsynaptic compartments. Through sensing neuronal activity and local molecular cues, microglia adjust their functional state and thereby modulate synaptic transmission and plasticity (Pereira-Iglesias et al., 2025; Tremblay et al., 2010). This pattern of dynamic contact not only provides a mechanistic basis for microglial detection of neuronal firing and molecular gradients such as ATP, but also constitutes a structural platform that enables the release of plasticity-regulating factors, including neurotrophic factors and cytokines (Miyamichi et al., 2011; Pinto et al., 2024).

Under physiological conditions, microglia sense neurotransmitter spillover and purinergic signals through such transient contacts and can release supportive factors, including BDNF, to maintain or refine synaptic connections. However, under NP conditions induced by peripheral nerve injury, microglia in the spinal dorsal horn (SDH) rapidly undergo morphological and functional state transitions, characterized by somatic hypertrophy, process remodeling, and proliferation, accompanied by pronounced changes in gene expression and immune functions (Malcangio et al., 2025). These pathologically activated microglia upregulate receptors and molecular regulatory programs associated with phagocytosis, for example, TREM2, which participates in the control of synaptic recognition and complement receptor pathways and exhibits an increased propensity for the internalization of synaptic components (Whalley, 2017). Experimental evidence indicates that following peripheral nerve injury, microglia selectively engulf specific types of synaptic structures within the SDH, such as inhibitory synapses, and participate in circuit remodeling, a process that is closely associated with the maintenance of pathological pain states (Gao et al., 2023).

### 2.3 Physical barrier role of the extracellular matrix within the synaptic microenvironment

In addition to cellular components, the synaptic microenvironment also contains a structurally complex extracellular matrix (ECM). In the spinal dorsal horn (SDH), the ECM is predominantly organized in the form of perineuronal nets (PNNs), which are densely distributed around fast-spiking inhibitory interneurons, such as Parvalbumin^+^ neurons (Totaro et al., 2025; Fawcett et al., 2022). PNNs consist of a hyaluronan backbone, link proteins, and chondroitin sulfate proteoglycans (CSPGs) rich in chondroitin sulfate, together forming a stable mesh-like structure (Fawcett et al., 2019).

PNNs play dual roles in maintaining synaptic homeostasis by providing both a physical barrier and ionic buffering functions. Their structural properties not only restrict the lateral diffusion of extrasynaptic receptors, thereby stabilizing synaptic connections, but also, owing to their high negative charge density, establish relatively confined immune microdomains surrounding synapses, reducing the accessibility of large immune complexes (Sorg et al., 2016; Carceller et al., 2023). Accordingly, the ECM not only provides structural support for synapses but also plays an important role in synaptic immune regulation. Under pathological conditions, the structural integrity of the ECM is often compromised prior to overt synaptic damage, and its degradation products can act as damage-associated molecular patterns (damage-associated molecular patterns, DAMPs), further activating glial cells and thereby exacerbating local inflammatory responses (Fawcett et al., 2019; Tewari et al., 2022).

Therefore, the synaptic microenvironment of the spinal dorsal horn (SDH) constitutes a dynamically regulated network jointly composed of neurons, glial cells, and the extracellular matrix (ECM). Peripheral nerve injury not only alters neuronal excitability but also remodels this microenvironment at the molecular and structural levels, thereby weakening the homeostatic protective mechanisms of inhibitory synapses and providing the necessary spatiotemporal conditions for the local initiation and action of the complement cascade within the SDH.

## 3 Complement cascade–mediated recognition and physical elimination of inhibitory synapses

**FIGURE 2:**
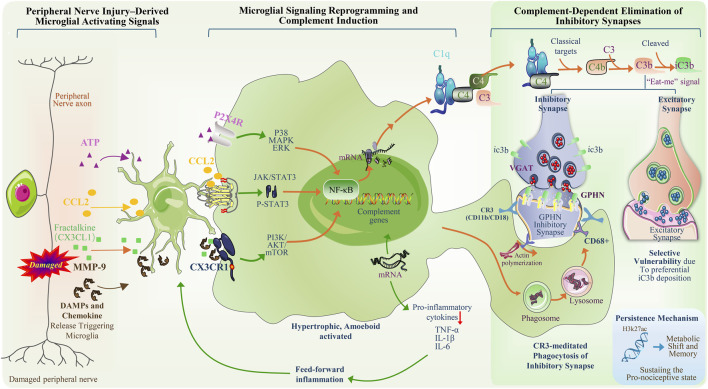
Schematic illustration of peripheral nerve injury–driven microglial signaling reprogramming and complement-dependent elimination of inhibitory synapses. Peripheral nerve injury–associated signals: peripheral axonal damage; release of ATP, CCL2, Fractalkine (CX3CL1), damage-associated molecular patterns (DAMPs), and matrix metalloproteinase MMP-9; transmission of sensory neuron–derived signals to the spinal dorsal horn. Microglial signaling pathway reprogramming: expression of receptors including P2X4R and CX3CR1; activation of signaling axes such as p38 MAPK/ERK, JAK/STAT3, and PI3K/AKT/mTOR; NF-κB–mediated transcriptional regulation; transcription of complement-related genes; expression of proinflammatory cytokines (TNF-α, IL-1β, IL-6); microglial morphological states (hypertrophic, amoeboid). Complement-dependent elimination of inhibitory synapses: C1q binding; activation of the classical complement pathway; cleavage of C4 and C3 and deposition of iC3b; comparison between inhibitory and excitatory synapses; inhibitory synaptic markers (VGAT, GPHN); CR3 (CD11b/CD18)–mediated recognition; actin polymerization; phagosome and lysosome structures; CD68^+^ phagocytic pathway; selective vulnerability of inhibitory synapses. Features associated with sustained mechanisms: epigenetic marks (H3K27ac); metabolic states and immune memory–related features; maintenance of a pro-nociceptive state. (Some graphical elements were adapted and modified from Servier Medical Art (https://smart.servier.com), licensed under a Creative Commons Attribution 3.0 Unported License).

The development of NP transforms the spinal dorsal horn (SDH) from a relay structure primarily dedicated to sensory transmission into a pathological processing unit involved in aberrant signal amplification, a transition that is closely associated with central sensitization and functional reorganization of local microcircuits (Todd, 2010; Tritsch et al., 2016). This functional shift does not arise from nonspecific inflammatory injury but instead predominantly reflects structural remodeling at the synaptic level, particularly the selective reduction of inhibitory synaptic connections (Peirs et al., 2015; Yousefpour et al., 2023). The complement cascade, a classical immune defense mechanism, can be aberrantly reactivated and functionally reprogrammed in the mature central nervous system under pathological conditions, thereby mediating selective actions on specific synaptic subpopulations (Stevens et al., 2007; Yousefpour et al., 2025). This process is not merely passive phagocytosis, but depends on the stable anchoring of complement molecules on synaptic surfaces and cytoskeletal remodeling mediated by microglial CR3 (complement receptor 3, integrin αMβ2, CD11b/CD18) (Jaques et al., 1996; Vorup-Jensen and Jensen, 2018); alterations in the physicochemical properties of the synaptic membrane may also contribute to the regulation of complement recognition. Accordingly, this process involves not simple phagocytic clearance, but multiple spatiotemporally coupled regulatory events, including changes in membrane lipid states, covalent anchoring of complement molecules, and cytoskeletal remodeling (Figure 2).

### 3.1 Mechanisms by which the C1q complex recognizes presynaptic membrane lipid nanodomains

As the initiating recognition molecule of the classical complement pathway, C1q is an approximately 460 kDa supramolecular protein complex composed of 18 polypeptide chains (six A chains, six B chains, and six C chains) assembled into a bouquet-like hexameric structure. Its N-terminal collagen-like stalks and C-terminal globular recognition domains together constitute a canonical pattern-recognition module (Gaboriaud et al., 2003; Son, 2022; Kishore and Ghebrehiwet, 2020). The six globular head domains of C1q are capable of recognizing a broad range of molecular patterns, including immune complexes, acute-phase proteins, and other surface ligands, thereby triggering activation of the classical complement cascade (Kishore et al., 2016).

Under physiological homeostasis, phospholipid asymmetry between the inner and outer leaflets of the plasma membrane is primarily maintained by ATP-dependent flippases such as P4-ATPases. These P4-ATPases establish an enrichment of phosphatidylserine (PS) on the inner leaflet by transporting PS and phosphatidylethanolamine (PE) from the outer to the inner leaflet, a process that is essential for lipid asymmetry of the presynaptic membrane (Andersen et al., 2016; No et al., 2024; He et al., 2020). Under pathological conditions, metabolic stress and alterations in intracellular Ca^2+^ levels may disrupt flippase activity and promote mechanisms of membrane lipid redistribution, whereas the Ca^2+^-dependent phospholipid scramblase TMEM16F can mediate PS externalization in response to elevated intracellular Ca^2+^ (Suzuki et al., 2010; Suzuki et al., 2013).

Disruption of membrane homeostasis can weaken phospholipid asymmetry and induce the exposure of phosphatidylserine (PS) on the outer leaflet of the plasma membrane, a process that is particularly pronounced under conditions of elevated Ca^2+^ and metabolic stress (Stevens et al., 2007; Hong et al., 2016). Studies have shown that externalized PS displays spatially heterogeneous distributions on the plasma membrane rather than uniform diffusion (Paolicelli et al., 2011). Within the complement system, C1q mediates multivalent ligand binding through its multimeric globular head structure, and the avidity generated by such multivalent interactions stabilizes the complex and induces conformational changes in the C1 complex, thereby relieving the autoinhibitory state of C1r_2_s_2_ and activating the C1s serine protease to initiate the classical complement cascade (Ghebrehiwet and Peerschke, 2004). Accordingly, disruption of synaptic membrane lipid homeostasis may, by altering local physicochemical surface properties, increase the spatial probability of multivalent complement binding and activation, thereby converting neuronal metabolic and electrical activity states into immune signals that can be recognized and amplified by the complement system.

### 3.2 Chemical properties of the C4 thioester bond and covalent anchoring of complement molecules

Genome-wide association studies (GWAS) have shown that structural variation in the C4 gene, particularly copy number variation of C4A, is associated with neuropsychiatric phenotypes such as schizophrenia, and that increased C4A expression in transgenic mouse models leads to enhanced microglia-mediated synaptic elimination and reduced synaptic density (Yilmaz et al., 2021). Following activation of the classical pathway, the C4 protein exposes an internal thioester bond; this reactive moiety, upon proteolytic cleavage, can form covalent bonds with amino or hydroxyl side chains on target surfaces, thereby stably anchoring complement activation signals to the local surface (Law and Dodds, 1997).

Following C1s-mediated proteolysis, the complement component C4 is cleaved into C4b and undergoes conformational rearrangement, thereby exposing a highly reactive internal thioester (TE) bond that is normally buried within the hydrophobic core (Law and Dodds, 1997). This TE bond exhibits low stability in an aqueous environment at physiological pH and has a very short half-life; if it does not react promptly, it is rapidly hydrolyzed and inactivated (Bowker-Kinley et al., 1998). Consequently, C4b must, within a very short time after its generation, diffuse to and contact adjacent cell membrane surfaces, allowing the TE bond to undergo nucleophilic reactions with hydroxyl or amino groups on membrane proteins or glycocalyx molecules to form stable covalent ester or amide bonds, thereby spatially confining complement activation signals to the local surface (Lea, 2002). Membrane-bound C4b can then efficiently recruit complement component C2 and, under the action of C1s, form the C3 convertase complex (C4b2a), enabling spatially localized amplification of the complement cascade (Walport, 2001). Thus, the transient covalent anchoring mechanism mediated by the internal thioester bond of C4b allows the complement cascade to convert fleeting recognition events into a highly spatially restricted and amplifiable enzymatic platform.

The C4b2a complex essentially constitutes an efficient enzymatic platform localized on the synaptic surface, capable of cleaving downstream C3 with high catalytic efficiency, thereby markedly amplifying the initial recognition signal (C1q binding) in a local manner and promoting extensive deposition of C3b/iC3b on the synaptic surface (Ricklin et al., 2016). Accordingly, the efficiency of covalent C4 binding largely determines the magnitude of subsequent synaptic elimination responses and serves as a critical molecular node linking the complement recognition phase to the execution phase.

### 3.3 CR3-mediated synaptic phagocytosis and cytoskeletal remodeling

**FIGURE 3:**
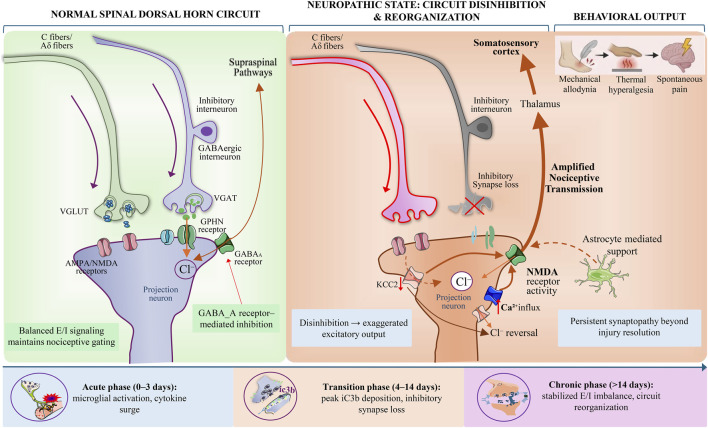
Schematic comparison of spinal dorsal horn circuit architecture and functional output under normal and neuropathic pain conditions. Normal spinal dorsal horn circuitry: inputs from C fibers and Aδ fibers; excitatory synaptic structures (VGLUT, AMPA/NMDA receptors); GABAergic inhibitory interneurons; inhibitory synaptic markers (VGAT, GPHN); GABA receptor–mediated inhibition; projection neuron output; excitatory–inhibitory (E/I) signal balance; nociceptive sensory gating state; spinal–supraspinal pathway connectivity. Circuit reorganization under neuropathic pain conditions: reduced function of inhibitory interneurons; loss of inhibitory synapses; downregulation of KCC2 expression; alterations in intracellular chloride homeostasis; reversal of Cl^−^ equilibrium potential; enhanced NMDA receptor activity; increased Ca^2+^ influx; amplified excitatory output of projection neurons; E/I imbalance; establishment of a disinhibited state; astrocyte-derived supportive signaling. (Some graphical elements were adapted and modified from Servier Medical Art (https://smart.servier.com), licensed under a Creative Commons Attribution 3.0 Unported License).

The ultimate outcome of the cascade reaction is the formation of a high-density opsonin coating on the surface of targeted synapses (Lea, 2002). iC3b, a degradation product of C3b, serves as the principal ligand for CR3 (integrin αMβ2, CD11b/CD18) expressed on the surface of microglia. Microglia-mediated synaptic elimination is not a purely phagocytic process, but rather represents a form of partial synaptic engulfment involving mechanical signal transduction, referred to as synaptic trogocytosis (Figure 3).

Under chemokine stimulation, CR3 receptors on the surface of SDH microglia undergo a conformational transition from a low-affinity bent state to a high-affinity extended state, a change that facilitates CR3 binding to iC3b on the synaptic surface and initiates outside-in signal transduction (Vorup-Jensen and Jensen, 2018). During this process, phosphorylation of Src family kinases and Syk kinase occurs rapidly, leading to the assembly of signaling complexes via adaptor proteins (Hadas et al., 2012). Subsequently, Rho GTPases such as Rac1 and Cdc42 are activated, promoting F-actin polymerization and driving outward membrane extension to form a phagocytic cup that envelops synaptic terminals (Caron and Hall, 1998). This process not only contributes to microglia-mediated synaptic elimination but also suggests that partial synaptic engulfment may represent an important feature of this mechanism (May and Machesky, 2001). Although “synaptic partial engulfment” currently lacks direct experimental validation, this phenomenon, as a speculative process, may occupy a role in synaptic clearance mechanisms.

During this process, the intracellular actomyosin system of microglia, particularly Myosin-II–mediated contractile forces, participates in phagocytic cup closure and membrane remodeling, providing the necessary mechanical support for the internalization of presynaptic structures (Swanson et al., 1999). Subsequently, endosomes containing synaptic components fuse with CD68^+^ lysosomes, where acidic hydrolases, such as Cathepsin D, mediate the degradation of presynaptic vesicle protein Synaptophysin and associated structural proteins (Hong et al., 2016; Schafer et al., 2012; Saftig and Klumperman, 2009). Through these steps, previously functional synaptic connections are structurally eliminated.

### 3.4 Multidimensional molecular mechanisms underlying the selective vulnerability of inhibitory synapses

In the context of widespread neuroinflammation associated with NP, the complement system exhibits pronounced selectivity toward inhibitory synapses, particularly GABAergic and glycinergic synaptic connections that collectively contribute to inhibitory gating in dorsal horn circuits, suggesting a systemic failure of defense mechanisms that are intrinsic to inhibitory neurons under pathological conditions. Based on the available evidence, this article proposes a “barrier–glycocode dual-conformity model” to explain the targeting specificity observed during complement-mediated synaptic elimination. Within this framework, structural protection provided by perineuronal nets and immune recognition signals encoded by the synaptic glycocalyx jointly determine the susceptibility of synapses to complement tagging. Rather than functioning as independent mechanisms, these two components may interact to shape the accessibility and recognition of inhibitory synapses by microglia.

#### 3.4.1 MMP-9–mediated proteolysis of perineuronal nets and physical exposure

PV^+^ inhibitory interneurons are typically ensheathed by perineuronal nets (PNNs) formed through the cross-linking of chondroitin sulfate proteoglycans (CSPGs), such as Aggrecan, with a hyaluronan backbone, a structure that plays a critical role in the stabilization and protection of inhibitory synapses (Sorg et al., 2016; Fawcett et al., 2019; Pantazopoulos et al., 2006). The dense lattice formed by PNNs may restrict the access of large complement molecules, such as C1q, to the synaptic membrane through steric hindrance (Tønnesen et al., 2023; Dityatev and Schachner, 2003; Kishore and Reid, 2000). However, during the early stages of nerve injury, matrix metalloproteinase-9 (MMP-9) released by microglia and astrocytes can specifically cleave the Aggrecan core protein, leading to rapid disruption of the PNN lattice structure. This degradation weakens the physical barrier surrounding synapses and increases the likelihood that inhibitory synapses become exposed to a complement-rich extracellular environment.

Therefore, PNNs protect inhibitory synapses through their physical barrier function; however, under conditions of nerve injury or inflammation, the release of MMP-9 leads to PNN disruption, thereby facilitating complement-mediated synaptic elimination and playing a critical role in the pathological processes that follow nervous system injury.

#### 3.4.2 Loss of immune checkpoint function caused by desialylation of the synaptic surface

Compared with the physical barrier function provided by PNNs, the synaptic surface glycocalyx plays a crucial molecular regulatory role in immune recognition (Puigdellívol et al., 2020). The surface of healthy neurons is enriched in terminal sialic acid residues, which can interact with Siglecs on the surface of microglia, such as Siglec-E, thereby recruiting SHP-1/2–containing phosphatases to inhibit ITAM (immunoreceptor tyrosine-based activation motif)–mediated phagocytic signaling pathways and maintain microglia in an inhibited state (Kukan et al., 2024; Claude et al., 2013).

Under pathological conditions, elevated levels of reactive oxygen species (reactive oxygen species, ROS) and glutamate spillover can induce aberrant activation or altered membrane localization of NEU on neuronal surfaces, particularly NEU1. NEU-mediated desialylation rapidly removes sialic acid residues from the synaptic surface, leading to attenuation of Siglec-E–dependent inhibitory signaling and a marked reduction in the activation threshold of microglia (Kukan et al., 2024; Nomura et al., 2017). Concurrently, desialylation exposes underlying galactose (Gal) or N-acetylgalactosamine (GalNAc) residues, which can be specifically recognized by endogenous lectins, such as Galectin-3. As a multivalent binding molecule, Galectin-3 can mediate cross-linking between C1q and the synaptic surface, forming stable molecular complexes that enhance local complement deposition and promote subsequent phagocytic processes (Puigdellívol et al., 2020; Radulova et al., 2024). Together, these molecular events indicate that the sialylation status of the synaptic glycocalyx is a key regulatory factor in suppressing microglial activation and complement-mediated phagocytosis, whereas the exposure of glycan epitopes following sialic acid loss may facilitate the binding of opsonins, including lectins and complement components, and promote microglia-mediated clearance responses.

#### 3.4.3 Metabolic load and susceptibility to oxidative stress in fast-spiking interneurons

The selective vulnerability of inhibitory neurons is thought to be closely associated with their distinctive metabolic characteristics. PV^+^ inhibitory interneurons are characterized by a fast-spiking firing pattern, accompanied by exceptionally high energy demands and a markedly increased mitochondrial density to sustain ATP production required for ion pump activity and high-frequency action potential firing (Hu et al., 2014; Kann et al., 2014). This metabolic profile renders PV^+^ neurons highly sensitive to mitochondrial dysfunction under injury- or inflammation-associated stress conditions and predisposes them to pronounced increases in ROS levels (Aizenman and Mastroberardino, 2015). Oxidative stress is considered to influence extracellular matrix homeostasis surrounding neurons through multiple indirect mechanisms, including enhancement of protease activity and reduced tolerance of PNNs to inflammatory and injury-related insults, thereby compromising their structural integrity (Cambiaghi et al., 2016). In this context, weakening of PNN structure may diminish the protective capacity of inhibitory synapses against pathological immune and complement signals. Concurrently, the reduction in inhibitory synapse number is regarded as the result of temporally and spatially coordinated actions of multiple pathological processes rather than a single molecular event. In the spinal dorsal horn, sustained loss of inhibitory synapses disrupts excitatory–inhibitory balance and promotes the establishment of a disinhibited state, which is considered one of the core mechanisms underlying the maintenance of chronic pain phenotypes (Chen and Tang, 2024; Hughes and Todd, 2020).

In summary, PV^+^ inhibitory neurons exhibit selective vulnerability under injury-associated stress conditions due to their high metabolic load. The accumulation of mitochondrial dysfunction and oxidative stress can synergistically promote inhibitory synapse loss by weakening the structural stability of PNNs and altering synaptic surface glycocode regulation, thereby driving the establishment of a disinhibited state in the spinal dorsal horn and contributing to the maintenance of chronic pain phenotypes.

## 4 Neuron–glia communication networks driving complement activation after nerve injury

**FIGURE 4:**
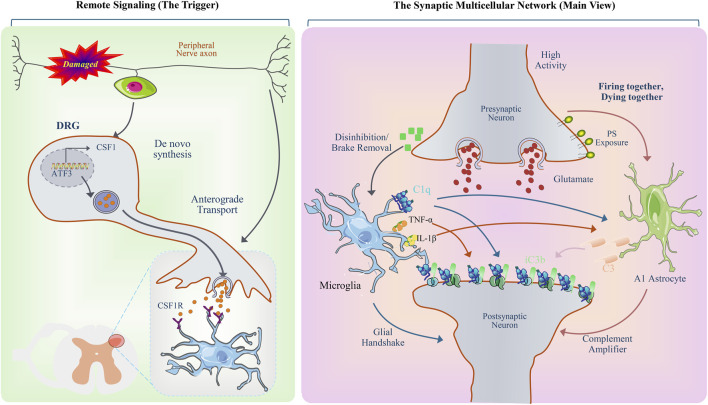
Schematic illustration of remote injury signal triggering and complement-related regulation within the synaptic multicellular network. Remote signaling module: peripheral nerve axonal injury; dorsal root ganglion (DRG) sensory neurons; ATF3 expression; *de novo* synthesis of CSF1; anterograde axonal transport; CSF1–CSF1R signaling axis in the spinal dorsal horn; microglial receptor expression and signal reception. Synaptic multicellular network module: high-frequency neuronal activity; presynaptic glutamate release; disinhibited state; phosphatidylserine (PS) externalization; microglial recruitment; C1q binding; release of proinflammatory cytokines (TNF-α, IL-1β); astrocytic A1 phenotype; complement components C3 and iC3b; complement deposition on the synaptic surface; neuron–glia “handshake” structures; postsynaptic neuronal architecture; complement signal amplification module. (Some graphical elements were adapted and modified from Servier Medical Art (https://smart.servier.com), licensed under a Creative Commons Attribution 3.0 Unported License).

Immune remodeling of the synaptic microenvironment is not driven by microglia alone, but rather arises from coordinated regulation through intercellular communication among neurons, microglia, and astrocytes following nerve injury (Paolicelli et al., 2011; Schafer et al., 2012; Allen et al., 2012). As a key effector pathway of synaptic pruning, activation of the complement cascade depends on neuron signal–induced complement tagging and is modulated by astrocyte-mediated microenvironmental regulation, thereby influencing microglial recognition and elimination of synapses (Stevens et al., 2007; Hong et al., 2016) (Figure 4).

### 4.1 Nerve injury–induced *de novo* synthesis of CSF1 and retrograde axonal transport

Pathological signals generated by peripheral nerve injury, such as nerve ligation or transection, can be retrogradely transmitted to the spinal dorsal horn (SDH) via the dorsal root ganglion (DRG) (Grosu et al., 2024). Traditional studies have primarily focused on the roles of classical neurotransmitters, including glutamate and substance P, in this process; however, recent transcriptomic and genetic studies indicate that *de novo* synthesis of CSF1 represents a key upstream event driving spinal microglial proliferation and activation following nerve injury (Guan et al., 2016).

Under physiological conditions, CSF1 expression levels in sensory neurons are extremely low; axonal injury markedly induces upregulation of the injury-responsive transcription factor ATF3, accompanied by a rapid increase in Csf1 transcription in injured DRG neurons (Guan et al., 2016; Holland and Ramer, 2023). Newly synthesized CSF1 protein can be transported along axons to the central projection zones of sensory neurons in the spinal dorsal horn, where it acts locally on microglia (Guan et al., 2016). The CSF1 receptor, CSF1R, is predominantly expressed on the surface of microglia in the central nervous system, and this spatial specificity of ligand–receptor distribution confers a high degree of cell-type selectivity to this signaling pathway (Zhou et al., 2025; Tarale and Alam, 2022). Binding of CSF1 to CSF1R activates DAP12-dependent signaling pathways within microglia, promoting cell cycle entry and inducing pronounced morphological and transcriptional phenotype changes (Guan et al., 2016; Yu et al., 2021; Du et al., 2025). Further studies have demonstrated that CSF1R signaling is required for injury-induced upregulation of multiple immune-related genes in microglia; sensory neuron–specific deletion of Csf1 or blockade of CSF1R at the spinal level significantly attenuates nerve injury–induced microglial responses and central immune activation (Guan et al., 2016). Accordingly, CSF1 is considered a critical upstream signaling molecule linking peripheral nerve injury to spinal immune responses.

### 4.2 Astrocytic A1 polarization and the synthesis and supply of complement components

Microglia-mediated synaptic elimination is not executed in isolation; astrocytes also play an essential cooperative regulatory role in this process (Liddelow et al., 2017; Lawrence et al., 2023). Classical neuroinflammation theory proposes that activated microglia can induce the conversion of quiescent astrocytes into a neurotoxic phenotype, namely, A1 astrocytes, through the secretion of factors such as IL-1β, TNF-α, and C1q (Lawrence et al., 2025).

The formation of A1 astrocytes has a significant impact on complement-mediated synaptic pruning. Although C1q is primarily derived from microglia, subsequent key components of the complement cascade, particularly C3 and C4, are mainly synthesized and secreted by A1 astrocytes (Liddelow et al., 2017; Barnum, 1995; Clarke et al., 2018).

In multiple NP models, reactive astrocytes in the spinal dorsal horn (SDH) exhibit marked upregulation of C3 expression. For example, in the spared nerve injury (SNI) mouse model, the population of C3^+^ astrocytes in the SDH is significantly increased, accompanied by elevated levels of proinflammatory cytokines such as IL-1α, TNF-α, and C1q (Liu et al., 2025). Moreover, in the chronic constriction injury (CCI) model, the increase in C3 levels within the SDH is primarily derived from astrocytes, whereas its receptor C3aR is broadly expressed on microglia and neurons, suggesting a regulatory role of astrocyte-derived C3 in the pathology of chronic pain (Mou et al., 2022; Peng et al., 2025). The resulting interglial interactions form a positive feedback regulatory loop: nerve injury–activated microglia release C1q together with proinflammatory factors to induce astrocytic polarization toward the A1 phenotype, while A1 astrocytes continuously supply C3/C4, thereby supporting microglia in executing complement-dependent synaptic opsonization and phagocytosis (Schafer et al., 2012).

In addition, A1 astrocytes can downregulate inhibitory molecules involved in synaptic phagocytosis, such as MEGF10, and release proinflammatory mediators exemplified by lipocalin-2 (Lcn2), thereby further impairing the blood–spinal cord barrier (BSCB) and promoting the recruitment of peripheral immune cells (Jung and Ryu, 2023; Wang et al., 2024; Lawren et al., 2023). Consequently, astrocytes enhance the local effects of complement-related immune responses through multiple convergent pathways in this process.

### 4.3 Downregulation of the fractalkine signaling pathway and release of microglial inhibition

Under normal physiological conditions, in addition to immune-activating signals, the central nervous system possesses multiple immunosuppressive mechanisms to prevent nonspecific complement-mediated attacks on functional synapses (Veerhuis et al., 2011; Ricklin et al., 2010). Among these, the chemokine CX3CL1 expressed by neurons and its receptor CX3CR1, which is predominantly expressed on microglia, constitute a critical inhibitory axis that maintains microglia in a resting/ramified surveillance state and suppresses the expression of proinflammatory factors (Paolicelli et al., 2011; Ransohoff, 2016). CX3CL1 is constitutively expressed on the neuronal membrane, whereas its receptor CX3CR1 is selectively expressed on microglia (Cardona et al., 2006). Under physiological conditions, sustained interactions between membrane-bound CX3CL1 and CX3CR1 contribute to the maintenance of microglial ramified morphology and surveillance status while inhibiting the production of proinflammatory mediators.

Following nerve injury, protease activity in the spinal dorsal horn, such as that of Cathepsin S, is increased, leading to cleavage of membrane-bound CX3CL1 into a soluble form (Clark et al., 2007; Clark and Malcangio, 2014). Although soluble CX3CL1 retains chemotactic effects on microglia, the loss of membrane-bound signaling weakens inhibitory regulation of microglial activity (Paolicelli et al., 2014). Concurrently, under conditions of high-frequency neuronal firing, neurons can transiently downregulate surface CX3CL1 expression, further reducing the efficacy of this inhibitory pathway (Cook et al., 2010; Wolf et al., 2017). The attenuation of these inhibitory regulatory mechanisms interacts with CSF1-mediated positive activation signals, thereby synergistically lowering the activation threshold for microglial initiation of the complement cascade.

### 4.4 Temporal specificity and activity-dependent tagging of complement-mediated synaptic elimination

Complement-mediated synaptic actions are not random but instead exhibit pronounced spatial and synapse-type selectivity under specific physiological or pathological contexts. Across multiple developmental and disease models, complement signaling has been shown to preferentially participate in the elimination of inhibitory synapses (Lui et al., 2016; Wang et al., 2020). At the same time, substantial evidence indicates that complement-dependent synaptic elimination is closely linked to neuronal electrical activity states, with synapses exhibiting reduced or aberrant activity being more prone to complement deposition and subsequent microglial clearance (Stevens et al., 2007; Hong et al., 2016; Schafer et al., 2012).

Excessive excitation of presynaptic neurons can lead to elevated extracellular K^+^ concentrations and glutamate spillover, which not only activates postsynaptic NMDA receptors but also engages metabotropic glutamate receptors (mGluRs) expressed on the surface of glial cells (Nicholson and Syková, 1998; Porter and McCarthy, 1997). Local states of heightened activity may mark synapses through two pathways: first, Ca^2+^ influx may increase the recognizability of the synaptic membrane to complement components by inducing phospholipid rearrangements, such as phosphatidylserine (PS) externalization; second, neuronal activity can regulate the release of C3 from astrocytes into specific synaptic microdomains (Liddelow et al., 2017; Bazargani and Attwell, 2016). These mechanisms can be regarded as a process analogous to activity-dependent synaptic tagging (Stevens et al., 2007; Schafer et al., 2012). Under pathological conditions, this process may represent an aberrant exploitation of activity-related synaptic plasticity mechanisms.

In summary, complement-dependent synaptic elimination is unlikely to be driven linearly by a single molecular event, but rather represents the integrated outcome of multiple regulatory factors, including CSF1-mediated remote activation signals, complement components supplied by astrocytes, attenuation of the CX3CL1 inhibitory pathway, and dynamic changes in synaptic activity states. A systematic analysis of this upstream regulatory network will contribute to a more comprehensive understanding of NP pathophysiology and provide a theoretical basis for exploring potential intervention strategies beyond direct complement inhibition.

## 5 Ion homeostasis disruption and failure of sensory gating resulting from synaptic loss

Complement-dependent synaptic elimination not only alters synapse number but also affects information-processing properties by remodeling local circuits within the SDH (Todd, 2010; Wang et al., 2020). In addition to synaptic structural changes, alterations in glutamate clearance mechanisms may further contribute to central sensitization. Astrocytic glutamate transporters, particularly EAAT1 and EAAT2, play a critical role in maintaining extracellular glutamate homeostasis, and reduced transporter activity has been reported in several models of chronic pain. Impaired glutamate uptake may enhance excitatory drive in dorsal horn circuits and thereby amplify the functional consequences of inhibitory synapse loss. Multiple studies indicate that complement-dependent microglial phagocytosis preferentially targets inhibitory synapses across various models, and their loss disrupts the local excitation–inhibition (E/I) balance, further leading to disturbances in ionic homeostasis and abnormal neuronal firing patterns (Coull et al., 2005; Prescott and Ratté, 2012; Woolf, 2011). These changes impair the ability of the SDH to filter sensory inputs and ultimately promote the development of central sensitization and aberrant pain phenotypes.

### 5.1 Structural basis of inhibitory synapse loss and disruption of excitatory/inhibitory (E/I) balance

Sensory information processing in the SDH relies on feedforward and feedback inhibitory microcircuits constructed by local interneurons (Todd, 2010; Peirs and Seal, 2016; Duan et al., 2014). Accumulating evidence indicates that complement-mediated microglial synaptic elimination can markedly disrupt this inhibitory architecture (Wang et al., 2020; Moore et al., 2002). Electrophysiological analyses show that, at early stages of NP, superficial SDH neurons (Lamina I–II) exhibit a predominant reduction in the frequency of mIPSCs, whereas changes in amplitude are minimal (Moore et al., 2002). Further quantal synaptic analyses suggest that this alteration mainly reflects a decrease in the number of functional synaptic release sites (N), rather than changes in the conductance of individual postsynaptic receptor channels (q) (Be et al., 1995).

A reduction in the number of inhibitory synapses attenuates the efficacy of shunting inhibition (Koch et al., 1982; Mitchell and Silver, 2003). Under normal conditions, activation of γ-aminobutyric acid (gamma-aminobutyric acid, GABA) or glycine receptors increases membrane conductance and lowers the postsynaptic input resistance, thereby constraining the impact of excitatory currents on membrane potential fluctuations (Farrant and Kaila, 2007). When inhibitory synapses surrounding the soma or the axon initial segment are eliminated, neuronal input resistance increases, rendering previously subthreshold excitatory postsynaptic potentials more likely to summate and trigger action potential firing (Pouille and Scanziani, 2001). Such alterations in passive membrane properties are considered one of the key biophysical substrates underlying central sensitization.

### 5.2 Ionic homeostasis remodeling and polarity reversal of GABAergic transmission

Beyond alterations in synaptic architecture, complement-related neuroimmune responses can also markedly affect ionic homeostasis in postsynaptic neurons (Grace et al., 2014; Ji et al., 2018). In the context of complement activation and microglial activation, microglia-derived BDNF acts on TrkB receptors expressed on the neuronal surface, inducing downregulation of KCC2 expression and its internalization from the plasma membrane, thereby disrupting neuronal chloride homeostasis. It should also be noted that KCC2 dysregulation may arise through multiple mechanisms that are not exclusively dependent on immune signaling, including neuronal activity–dependent pathways and transcriptional regulation within dorsal horn neurons (Coull et al., 2005; Rivera et al., 2002). KCC2 is a key transporter responsible for maintaining a low intracellular [Cl^−^] concentration in mature neurons; impairment of its function leads to an elevation of intracellular Cl^−^ levels and a depolarizing shift of the Cl^−^ Nernst potential (Kaila et al., 2014; Ben-Ari et al., 2007; Medina et al., 2014).

When the Cl^−^ Nernst potential (E_Cl) becomes depolarized and exceeds the neuronal resting membrane potential, the direction of Cl^−^ flux through open GABA_A receptor channels is reversed, causing GABAergic transmission to become less hyperpolarizing and, under certain conditions, potentially depolarizing, a phenomenon referred to as the excitatory conversion of GABAergic signaling (Kaila et al., 2014; Ben-Ari et al., 2007; Price and Prescott, 2015). Under this condition, inhibitory inputs that normally constrain neuronal excitability instead promote membrane depolarization and can reinforce neuronal firing through a positive feedback process, thereby sustaining persistent activation of nociceptive transmission neurons and the state of central sensitization (Coull et al., 2005; Latremoliere and Woolf, 2009).

### 5.3 Failure of sensory gating and aberrant circuit recruitment

Under normal physiological conditions, the SDH selectively filters different sensory modalities through gating mechanisms, particularly restricting non-nociceptive tactile signals conveyed by Aβ fibers from accessing nociceptive pathways (Todd, 2010; Peirs and Seal, 2016). Anatomical and functional studies have shown that Aβ fibers can directly excite PKCγ interneurons located in the inner layer of lamina II, whereas under physiological conditions this pathway is subject to feedforward inhibition mediated by PV^+^ inhibitory interneurons, thereby preventing the activation of nociceptive projection neurons (Duan et al., 2014; Petitjean et al., 2015).

Under NP conditions, complement-associated immune responses can mediate the degradation of PNNs and promote the elimination of inhibitory synapses, thereby weakening feedforward inhibition onto PKCγ^+^ interneurons and allowing tactile inputs to aberrantly activate pain-related circuits (Duan et al., 2014; Carulli et al., 2010). This circuit reorganization leads to the misencoding of non-noxious stimuli as pain signals, which behaviorally manifests as touch-evoked pain (Peirs and Seal, 2016; Petitjean et al., 2015). Concurrently, reduced connectivity within inhibitory networks disrupts synchronized oscillatory activity in the spinal dorsal horn, induces aberrant firing of projection neurons, and compromises the capacity of supraspinal centers to exert descending modulation over pain signals (Baliki and Apkarian, 2015; Ossipov et al., 2014).

Taken together, the link between complement-mediated molecular regulation and clinical pain phenotypes likely involves a series of interconnected biophysical processes. Among these, the synergistic interaction between structural synapse loss (quantal reduction) and functional reversal of ionic gradients (KCC2 downregulation) is thought to compromise inhibitory gating in the SDH, thereby facilitating the aberrant entry of tactile signals into nociceptive pathways and promoting the maintenance of central sensitization (Arendt-Nielsen et al., 2018). This circuit-level perspective helps explain why pharmacological strategies that solely target neuronal excitability show limited efficacy in a subset of patients and suggests that chronic pain may be accompanied by deeper alterations in network structure and function.

### 5.4 Therapeutic implications and potential intervention strategies

Beyond mechanistic insights, understanding complement-mediated synaptic remodeling also provides a conceptual framework for identifying potential therapeutic targets in neuropathic pain. Several potential strategies may emerge from this model. One approach involves limiting complement activation within the spinal dorsal horn. Experimental studies have suggested that inhibition of early complement components such as C1q or C3 may reduce microglia-mediated synaptic elimination and preserve inhibitory circuit integrity. A second strategy may involve stabilizing the synaptic microenvironment. For example, preservation of perineuronal net integrity or modulation of extracellular matrix remodeling may help maintain the structural protection of inhibitory synapses. In addition, interventions targeting neuron–glia signaling pathways that regulate complement expression, including CSF1-CSF1R signaling or astrocyte-derived inflammatory mediators, may represent upstream approaches to limiting pathological immune activation in the spinal dorsal horn. Finally, therapies aimed at restoring ionic homeostasis, such as pharmacological enhancement of KCC2 function, may help counteract the functional consequences of inhibitory synapse loss. Although most of these strategies remain at a preclinical stage, they highlight potential translational avenues for disease-modifying interventions in neuropathic pain.

## 6 Conclusion

Taken together, the development and persistence of NP are not solely attributable to aberrant increases in neuronal excitability but are likely to involve systematic structural and functional remodeling of SDH microcircuits. The multi-level evidence integrated here suggests that aberrant reactivation of the complement system in the mature central nervous system may preferentially target inhibitory synapses and their associated microenvironmental structures through glial cell–mediated synaptic elimination, thereby disrupting excitatory–inhibitory balance and weakening sensory gating. Complement cascade activation, PNN degradation, synaptic glycocalyx remodeling, and ionic homeostasis imbalance together constitute an interlinked pathological network that provides an important circuit-level basis for the initiation and maintenance of central sensitization and aberrant pain phenotypes.

Based on these considerations, effective therapeutic strategies for NP may need to shift from approaches that solely suppress neuronal firing toward disease-modifying concepts targeting synaptic architecture and the immune microenvironment. Limiting complement-mediated synaptic elimination, preserving the structural integrity of inhibitory synapses, or restoring immune homeostasis between neurons and glial cells may help intervene at an upstream level in the process of pathological circuit remodeling. Further elucidation of synapse-protective mechanisms will not only deepen the understanding of chronic pain pathogenesis but also provide an important theoretical foundation for the development of novel analgesic strategies with sustained efficacy.
